# Response to Comments of Peter G. Mantle

**DOI:** 10.3390/toxins2102337

**Published:** 2010-09-29

**Authors:** Gary G. Schwartz, Richard A. Manderville, Annie Pfohl-Leszkowicz

**Affiliations:** 1 Departments of Cancer Biology, Urology, and Epidemiology and Prevention, Wake Forest University, Winston-Salem, NC 27157, USA; 2 Department of Chemistry and Toxicology, University of Guelph, Guelph, Ontario, N1G 2W1, Canada; Email: rmanderv@uoguelph.ca; 3 University of Toulouse, Laboratory Chemical Engineering, Department Bioprocess & Microbial System, UMR CNRS/INPT/UPS 5503, ENSA Toulouse, 1 avenue de l’Agrobiopôle, BP 32607, 31326, Auzeville-Tolosane, France; Email: leszkowicz@ensat.fr

**Keywords:** ochratoxin, testicular cancer, DNA adduct

## Abstract

The apparently high yield of testis tumors (25%) in rats exposed long-term to Ochratoxin A (OTA) is uninterpretable without data on tumor yield in unexposed rats. Conversely, our demonstration that prenatal exposure to OTA induces DNA adducts in the testes of newborn mice and the absence of these adducts in the testes of mice not exposed prenatally to OTA, is evidence for the presumptive carcinogenicity of OTA in the testis. Together with recent data showing that prenatal exposure to OTA depresses expression of DMRT1, a tumor suppressor gene in the testis, our findings suggest that OTA may be a cause of testicular cancer.

We thank Pr. Mantle for his comments about our paper [1]. In his commentary, Pr. Mantle refers to perceived mis-citations of his papers. In particular, we cited his recent observation that 6/24 (25%) of aged Fisher rats exposed to Ochratoxin A via the diet developed testicular tumors [2]. We interpreted this as *a priori* evidence of a tumorigenic effect of Ochratoxin A on the testis, although that paper had no control group. We also noted his 2005 paper which also reported testicular tumors in rats exposed to Ochratoxin A [3]. Pr. Mantle writes in his commentary that, “it was clearly stated there [the 2005 paper] that testis tumours occurred equally in treated rats and in controls”. However, careful review of that paper with regard to testis tumors reveals only this comment: “*In some older animals from both control and treated groups, single or multiple seminomas occurred within one or both testes* (p. 61)”. Thus, absent data from a control group [2], and absent data on the incidence, multiplicity and histology of tumors from an experimental and control group [3], it would be more accurate to conclude that no inference about the role of Ochratoxin A in testicular tumors can be derived from the papers of Mantle *et al.*

In contrast, our observations of DNA adducts in the testes of mice exposed prenatally to Ochratoxin A, and the absence of any such adducts in the testes of control mice not exposed prenatally to Ochratoxin A, are clear evidence of the carcinogenic potential of Ochratoxin A in the testes. The DNA adducts that we observed are not evidence merely of exposure (as Pr. Mantle suggests), they are markers of **biological effect**, as DNA adducts are widely considered to be markers of an increased risk of cancer [4,5,6]. Furthermore, we note that prenatal exposure to Ochratoxin A in mice significantly depresses expression of the *DMRT1* gene in offspring, particularly male offspring [7]. *DMRT1* is a doublesex and mab-3 related transcription factor that is expressed in Sertoli cells and undifferentiated spermatogonia of the postnatal testis [8]. *DMRT1* is a tumor suppressor gene in the testis; loss of this gene produces germ cell testicular tumors in mice [9]. A large genome-wide study from the United Kingdom recently confirmed a role for *DMRT1* in testicular germ cell tumors in humans [10]. Thus, in addition to our study, considerable molecular evidence supports the hypothesis that Ochratoxin A may be causally related to germ cell testicular tumors in mice and in men [11]. 
